# Three-Dimensional Surgical Planning in Mandibular Cancer: A Decade of Clinical Experience and Outcomes

**DOI:** 10.3390/cancers18020271

**Published:** 2026-01-15

**Authors:** Li H. Yang, Bram B. J. Merema, Joep Kraeima, Koos Boeve, Kees-Pieter Schepman, Marijn A. Huijing, Eva S. J. van der Beek, Martin W. Stenekes, Jeroen Vister, Sebastiaan A. H. J. de Visscher, Max J. H. Witjes

**Affiliations:** 1 Department of Oral & Maxillofacial Surgery, University Medical Center Groningen, University of Groningen, Hanzeplein 1, 9700 RB Groningen, The Netherlandss.a.h.j.de.visscher@umcg.nl (S.A.H.J.d.V.); 2 3D-Laboratory, University Medical Center Groningen, University of Groningen, 9713 GZ Groningen, The Netherlands; b.j.merema@umcg.nl (B.B.J.M.); j.kraeima@umcg.nl (J.K.); 3 Department of Plastic & Reconstructive Surgery, University Medical Center Groningen, University of Groningen, Hanzeplein 1, 9700 RB Groningen, The Netherlands; 4 Department of Radiology, University Medical Center Groningen, University of Groningen, Hanzeplein 1, 9700 RB Groningen, The Netherlands

**Keywords:** oral squamous cell carcinoma, three-dimensional virtual surgical planning, mandibular reconstruction, care pathway interval, patient-specific implants, surgical accuracy

## Abstract

**Background:** Three-dimensional virtual surgical planning (Three-dimensional VSP) has become standard practice in the treatment of mandibular oral squamous cell carcinoma (OSCC) in the last decade. Dutch guidelines recommend a care pathway interval (CPI) of a maximum of 30 days, and a free bone margin of at least 5 mm. Fused MRI and CT data are used for accurate tumor delineation. Based on this data, a virtual surgical plan is created and transferred to the operating room using resection guides and patient-specific implants (PSIs). Long-term evaluation is needed to further optimize its clinical use. **Objectives:** This study evaluates adherence to bone margin and CPI guidelines in mandibular OSCC. Additionally, it assesses the accuracy of tumor resection and reconstruction using 3D-VSP and compares the complications of 3D-planned mandibular reconstruction using different kinds of osteosynthesis plates. **Methods:** All patients who underwent a segmental mandibulectomy between 2014 and 2024 at the University Medical Center Groningen were included. CPI, clinical outcomes, and complications were analyzed. The preoperative virtual plan was compared with the postoperative outcome to assess accuracy. **Results:** The median CPI was 34 days, and 93.7% of bone margins were tumor-free. Mean absolute resection deviation was 1.63 mm (±1.42). PSI reconstructions were significantly more accurate in intergonial distance and coronal angle compared to conventional plates. Plate-related complications were more common in non-bony reconstructions; PSI reconstructions showed significantly more plate exposure. **Conclusions:** 3D-VSP leads to high accuracy in resection and reconstruction and favorable bone margins. Shortening the CPI and reducing biological complications are essential to further improve oncological outcomes.

## Simple Summary

Cancer of the mouth often requires removal of part of the jaw. Dutch guidelines recommend surgical treatment within 30 days and the achievement of tumor-free bone margins of >5 mm. Three-dimensional virtual surgical planning has become standard practice in treatment planning as it improves accuracy and predictability of resection and reconstruction. Resections are performed using resection guides, followed by reconstruction with either hand-bent plates or 3D-designed implants. This study evaluates guideline adherence, plate-related complications, and the accuracy of 3D-planned mandibular resection and reconstruction using different osteosynthesis plates. The median time to surgery in this cohort slightly exceeded the guideline recommendations, and a high percentage of bone margins were tumor-free. Bridging the mandibular gap with just a reconstruction plate showed higher plate-related complications compared to reconstructions in which bone could be inserted. Resection was performed with high accuracy. Reconstructions with 3D-designed implants were more accurate in selected dimensions than conventional plates.

## 1. Introduction

Treatment of oral squamous cell carcinoma (OSCC) often requires segmental mandibulectomy when it is located close to, or invading, the mandible [1]. Additionally, Dutch clinical guidelines recommend that surgery is performed within 30 days of the initial consultation, a period referred to as the care pathway interval (CPI) [2]. To minimize the risk of local recurrence, current international and Dutch clinical guidelines recommend achieving a microscopic bone margin of at least 5 mm [3]. Both delay in treatment and inadequate margins have been associated with tumor progression and reduced overall survival, making adherence to both timing and margin guidelines crucial in head and neck cancer management [2,3,4,5,6].

To meet these oncological and surgical requirements, three-dimensional virtual surgical planning (3D-VSP) has been routinely implemented in the treatment planning of mandibular squamous cell carcinoma in the University Medical Center Groningen, the Netherlands, (UMCG) over the past decade. While it may increase the preoperative workload, 3D-VSP improves the accuracy, predictability, and safety of both tumor resection and reconstruction [7]. Successful 3D-VSP depends on accurate preoperative tumor delineation. Preoperative imaging, including CT and MRI, provides detailed structural information. CT excels at visualizing bone structures, while MRI offers superior soft-tissue detail, required for tumor delineation [1].

The fusion of both modalities provides a more precise representation of the tumor and improves surgical accuracy [1]. This information is used to create a virtual model of the mandible and tumor to simulate resection and reconstruction. Based on this virtual surgical plan, 3D-printed surgical guides and either an anatomical 3D-printed model for manual pre-bending or patient-specific implants (PSIs) can be designed and manufactured preoperatively. These are then applied during surgery to ensure adherence to the plan and accuracy [7].

Following resection, osteosynthesis plates preferably secure a free vascularized graft in place for mandibular reconstruction [8]. Traditionally, conventional plates require manual bending during surgery to achieve a proper fit. This method poses drawbacks, as the bending process induces internal residual stress, weakens the plate, and makes the outcome of the procedure less predictable [9]. To address these limitations, patient-specific implants (PSIs) were developed. PSIs are designed according to the patient’s anatomy and precisely milled or additively manufactured to match the bone contour, avoiding the need for intraoperative manual bending [8]. This development reduces operation time and improves surgical accuracy, allowing for precise translation of the virtual plan into the surgical procedure [9]. More recently, modified PSIs have been developed for use without the need for a free vascularized bone graft [8].

Various plate-related complications can arise following mandibular reconstruction, some of which may affect clinical outcome. Plate exposure is one of the most common complications, with an occurrence of 11–30% in the literature [10,11,12,13,14,15,16,17]. Bone resorption with subsequent screw loosening is another well-recognized problem. This can be the result of stress shielding, causing the bone to locally lose its bone formation stimulus [8,18,19]. In addition, plate fracture may occur [10,11,12,13,14,15,16,17].

As both delay in treatment and inadequate margins are linked to poorer oncologic outcomes, adherence to clinical guidelines, including the achievement of safe bone margins and timely intervention within the crucial 30-day period, must be critically examined to ensure the highest standard of patient care.

It is also important to investigate whether PSIs result in improved outcomes compared to conventional plates, as plate-related complications may necessitate revision surgery and plate removal [10,11,12,13,14,15,16,17]. Long term evidence of resection and reconstruction accuracy using PSIs versus conventional plating is also lacking. Such studies are needed to evaluate the accuracy of resection and reconstruction and to optimize the use of PSIs in clinical practice.

Therefore, this study aims to evaluate adherence to clinical guidelines by analyzing both the timeframe between the first consultation and surgical treatment and the adequacy of bone margins. In addition, the study compares the complications of 3D-planned mandibular reconstruction using different kinds of osteosynthesis plates. Finally, it evaluates the accuracy of mandibular tumor resection and reconstruction using 3D-VSP with multimodal imaging as part of routine clinical care. The goal is to validate and expand current evidence to improve patient care.

## 2. Materials and Methods

### 2.1. Clinical Workflow

**Figure 1:**
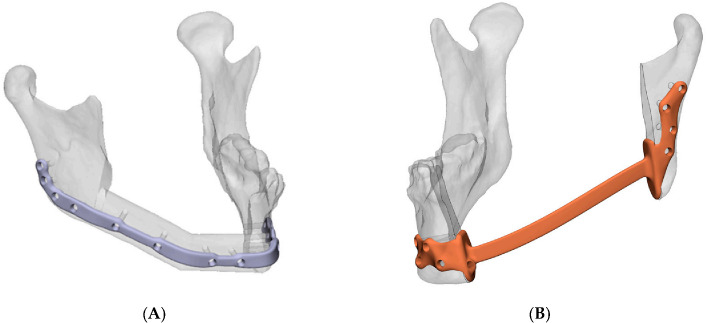
(**A**) Patient-specific implant; (**B**) Modified patient-specific implant, Bookshelf-PSI.

As part of the diagnostic workup, all patients received both CT and MRI imaging for tumor assessment and delineation. This imaging data was used to create a 3D-VSP, which was used to design either a PSI or an anatomical 3D-printed model for manual pre-bending of conventional osteosynthesis plates. In general, when a single fibula segment is needed to bridge the mandibular gaps, hand-bent osteosynthesis plates are used. Multiple fibula segments are planned in combination with a PSI. Two types of PSI were used, one which was used with bone grafts and one which was used without a bone graft, the Bookshelf-PSI (see Figure 1). In all cases, resections were performed using cutting guides, irrespective of which osteosynthesis plate was used. After tumor resection, histopathological assessment was performed, and postoperative imaging was obtained using either CBCT, or, when not available, CT. Patients underwent routine clinical follow-up, and in certain cases, patients received adjuvant radiotherapy, sometimes combined with chemotherapy.

### 2.2. Study Design and Population

Approval for this retrospective review was obtained from our institutional ethical review board with research registration number: 22457. All patients who underwent a primary segmental mandibulectomy with 3D-planned resection and reconstruction for oral squamous cell carcinoma at the Department of Oral and Maxillofacial Surgery of the UMCG between 2014 and 2024 were identified through the hospital’s electronic patient record system. All patients were included in the analysis of the CPI, clinical outcomes, and complication rates. For the accuracy analysis, patients were required to have both CT and MRI imaging as part of the diagnostic workup, as well as tumor volume delineation for preoperative planning. Patients were excluded from the accuracy analysis if data related to the 3D-VSP or imaging were missing, or if quality was insufficient for analysis. Postoperative imaging, which is used to determine accuracy, should be performed before radiotherapy is started. Cases without postoperative imaging were excluded, as accuracy analysis could not be performed.

### 2.3. Data Acquisition

Demographics and clinical data were collected, to assess CPI, clinical outcome, and complication rate. The Brown classification was used to describe defect size [20]. Imaging data from the virtual surgical plan were compared with postoperative results to evaluate surgical accuracy and outcomes. Postoperative CT or CBCT images were exported as Digital Imaging and Communications in Medicine (DICOM) files and imported into Mimics software 27.0 (Materialise, Leuven, Belgium) for segmentation. STL files of the segmented skull, right and left segments of the mandible, and the graft and resection planes were imported into 3-Matic software 19.0 (Materialise, Leuven, Belgium) for further analysis.

### 2.4. Orientation

**Figure 2:**
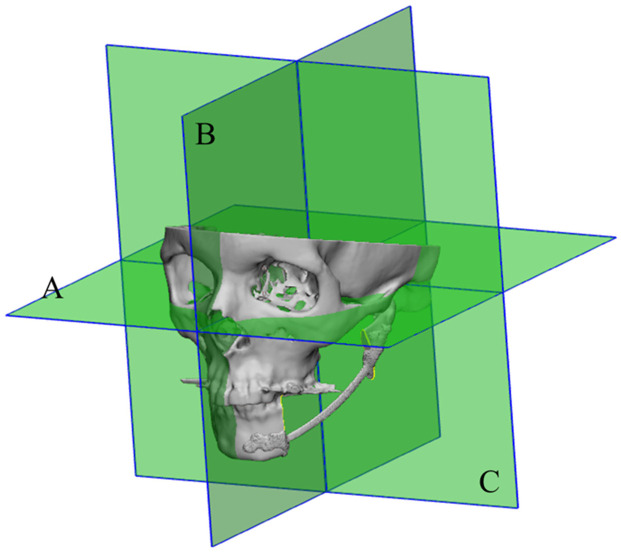
Graphic showing the orientation planes A: Frankfort horizontal plane; B: Midsagittal plane; C: Coronal plane.

In 3-Matic, the XYZ orientation was established by defining the Frankfort horizontal midsagittal and coronal planes, constructed using standard anatomical landmarks. The Frankfort horizontal plane connected the superior borders of the external acoustic meatuses and the right inferior orbital rim [21]. The midsagittal plane was created by rotating a duplicate of the Frankfort plane 90 degrees to pass through the nasion and basion of the skull [22]. The coronal plane was constructed by rotating the midsagittal plane 90 degrees in the coronal direction (see Figure 2). This method ensures consistent orientation between the preoperative and postoperative imaging data.

### 2.5. Resection Plane Accuracy Analysis

**Figure 3:**
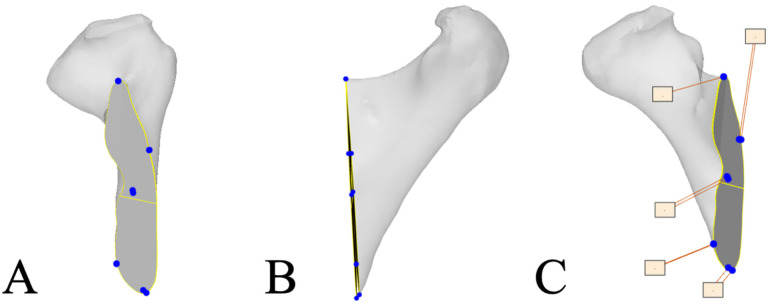
Visualization of resection plane accuracy measurements: (**A**) Frontal view of resection plane with marked reference points (Blue dots); (**B**) Lateral view showing deviation of the preoperative and postoperative planes; (**C**) Measurement of the deviation between preoperative and postoperative resection planes, in which the difference between the blue dots was calculated.

To evaluate resection accuracy, the segmented postoperative mandible and the corresponding resection planes were aligned with the preoperative 3D-VSP using the iterative closest point principle [23]. For both anterior and posterior resection planes, five key points were identified: the superior, inferior, lingual, and buccal points, along with the point of the center of gravity. The spatial distances between these points were calculated as the Euclidean difference between landmarks. Additionally, the angular deviation between the planned and actual resection planes was measured to assess rotational inaccuracies (see Figure 3).

### 2.6. Mandibular Reconstruction Accuracy Analysis

**Figure 4:**
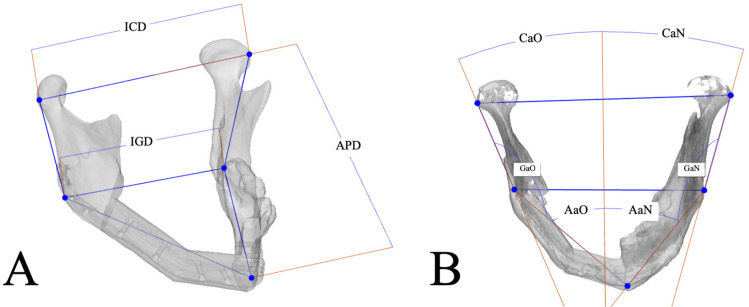
Visualization of linear and angular measurements: (**A**) ICD = intercondylar distance; IGD = intergonial distance; APD = anteroposterior distance. (**B**) CaO = Coronal angle of the operated side; CaN = Coronal angle of the non-operated side; GaO = Gonial angle of the operated side; GaN = Gonial angle of the non-operated side; AaO = Axial angle of the operated side; AaN = axial angle of the non-operated side.

Reconstruction accuracy was assessed by comparing the preoperative 3D-VSP with the postoperative result using linear and angular cephalometric measurements in 3-Matic software 19.0 for both models. Five key anatomical landmarks were identified: the most lateral point of each condyle, both gonion points and the pogonion. To ensure consistency and objectivity, these landmarks were defined using the reference planes. The lateral border of each condyle was defined by placing a duplicate of the midsagittal plane in contact with the lateral aspect of the condylar head [24]. The pogonion was defined by placing a duplicate of the coronal plane at the most anterior point of the mandible [24]. The gonion was defined as the point where a bisecting plane, constructed from planes along the posterior and inferior borders of the mandible, intersected the mandibular surface [25]. Linear measurements included the intercondylar distance (ICD), defined as the distance between both lateral borders of the condyles. The intergonial distance (IGD) was measured between the left and right gonion points. The anteroposterior distance (APD) was calculated from the pogonion and the intercondylar line (see Figure 4A). Angular measurements included the coronal angle, calculated as the angle between the line created from the lateral condyle and the gonion and the midsagittal plane. For the axial angle, a line connecting the gonion and the pogonion was created for both sides, and its angle to the midsagittal plane was measured. The gonial angle was determined by using the planes along the posterior and inferior borders (see Figure 4B).

### 2.7. Statistics

Data analysis was performed using IBM SPSS statistics version 30 (IBM Corp., Armonk, NY, USA). Data normality was tested using the Shapiro–Wilk test. Continuous variables were summarized as mean ± SD or median with interquartile range (IQR), depending on distribution. Categorical data were reported as counts and percentages. Between- and within-group comparisons used *t*-tests or non-parametric alternatives. Categorical variables were compared using chi-square tests or Fisher’s exact tests. Correlation was assessed using Pearson’s or Spearman’s correlation, and predictive relationships were examined with linear regression. Agreement was evaluated using Cohen’s kappa, weighted where applicable. A two-tailed *p*-value < 0.05 indicates statistical significance. Intra- and inter-observer variability was supported by an intraclass correlation coefficient (ICC) calculation, with ≥0.75–1.00 as excellent [1].

## 3. Results

### 3.1. Study Population

In total, 111 patients underwent a primary segmental mandibulectomy with 3D-planned resection and reconstruction for oral squamous cell carcinoma at the Department of Oral and Maxillofacial Surgery of the UMCG between 2014 and 2024. One patient did not receive a reconstruction plate and was not included for analysis of complication rates. In 18 patients the 3D planning files were inaccessible. Seven patients missed MRI data, and four patients lacked a postoperative CT-scan. As a result, accuracy analysis could be performed on 82 patients. In the subset with complete data, specific analyses were performed using 111 patients.

### 3.2. Clinical Outcomes

The mean age was 66.7 years (SD ± 13.9), with most being female patients (54.1%). Sixty-nine (62.2%) patients were active smokers or had a history of smoking. As for alcohol use, this was the case in 55 patients (49.5%). Most tumors were staged clinically (71.2%) and pathologically (68.5%) as T4. Brown class II mandibular defects were most frequently seen (44.1%). The vascularized fibula graft was the main method for reconstruction (73.0%). In 29 cases (26.1%) no bone graft was used, either due to poor vascularization or the patient’s preference. Postoperatively, 71 patients (64.0%) received radiotherapy, and 19 patients (17.1%) received concomitant chemoradiation. The mean follow-up duration was 59.8 months (SD ± 144.8) (see Table 1). Postoperative scans were primarily CBCT (75.6%) with a slice thickness ranging from 0.2 mm to 1.0 mm. When no postoperative CBCT was available, CT scans planned for adjuvant radiotherapy (24.4%) were used for accuracy determination instead, these had a higher slice thickness of 2.0 mm.

### 3.3. Treatment Timelines

**Figure 5:**
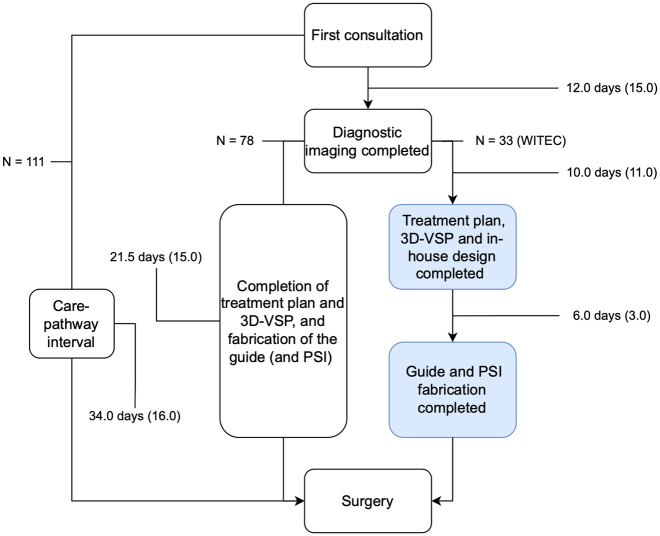
Flowchart of treatment timelines. Days are reported in median (IQR). Information about fabrication lead times were available for patient-specific implants. The in-house production by UMCG 3D-LAB is described on the right side (blue).

Focusing on treatment timelines, the median time from first consultation to completion of the diagnostic workup was 12.0 days (IQR 15.0). The median interval between completion of the diagnostic workup and surgery was 21.5 days (IQR 12.0). Overall, the median time from first consultation to surgery was 34.0 days (IQR 16.0), with 43 patients (38.7%) receiving treatment within 30 days. Information on the fabrication lead times for the resection guides and the PSI was not available for all cases; complete data was available for 33 patients. In these cases, the median interval between completion of the diagnostic workup and order date of the resection guides and PSIs was 10 days (IQR 11.0). The median fabrication time for the resection guides and PSIs was 6.0 days (IQR 3.0) (see Figure 5).

### 3.4. Margins

**Figure 6:**
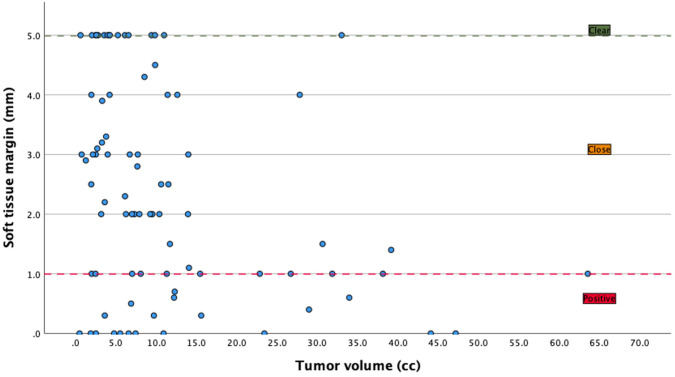
Graph illustrating that obtaining a clear margin in high volume tumors (>15.0 cc) is challenging.

Microscopic bone margins were >5 mm in 104 (93.7%) patients, while soft tissue margins were clear (>5 mm) in 28 (25.2%), close (1–5 mm) in 52 (46.8%) and positive (<1 mm) in 31 (27.9%). The mean tumor volume was 11.2 cc (SD ± 12.0) (see Table 2). Larger tumor volume was significantly associated with closer soft tissue margins, as demonstrated by a negative correlation (*ρ* = −0.317, *p* = 0.003) and confirmed by linear regression (B = −0.048, *p* = 0.002) (see Figure 6).

### 3.5. Complication Rates

#### 3.5.1. Plate-Related Complications

**Figure 7:**
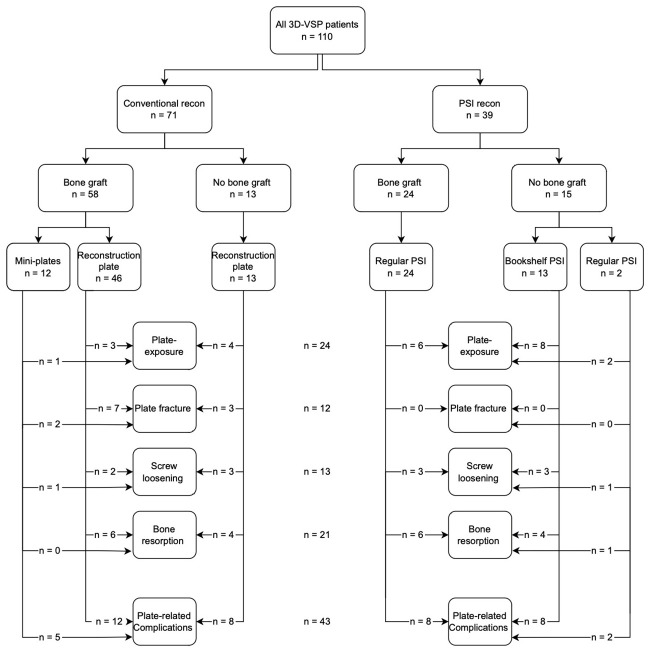
Detailed flowchart illustrating the different plate-related complications of the cohort. In the chart the use of conventional plates and PSIs in bone and non-bony reconstructions are separated.

Plate-related complications, defined as the presence of one or more of the following complications: plate exposure, plate fracture, bone resorption, or screw loosening, were observed in 43 of 110 patients (39.1%). The absence of a bone graft was significantly associated with a higher plate-related complication rate (64.2% vs. 30.5%, *p* = 0.002). Multi-segment bone reconstructions (*n* = 33/82) revealed a higher plate-related complication rate compared to single-segment bone reconstructions (*n* = 49/82); this difference was not statistically significant. (33.3% vs. 28.6%, *p* = 0.646). Complication rates, according to the Brown classification, increased with the severity of the defect: 31.6% in class I, 39.6% in class II, and 52.2% in class III. No significant difference in plate-related complications was found between reconstructions with bone using the conventional plate and PSI. (26.1% vs. 33.3%, *p* = 0.524). When comparing plate-related complications in reconstructions without bone, no significant differences in frequency were reported. (61.5% vs. 61.5%, *p* = 1.00) (see Figure 7).

#### 3.5.2. Plate Exposure

The most frequent complication was plate exposure occurring in 24 of the 110 patients (21.8%). Fifteen of the affected patients had received postoperative radiotherapy. In 12 patients the plate was removed due to plate exposure. Plate exposure was significantly more common in patients without a bone graft (12.2% vs. 50%, *p* < 0.001). Plate exposure was notably more common in patients with a Brown class III defect (39.1%) compared to class I (10.5%, *p* = 0.008) and class II (16.7%, *p* = 0.155). Multi-segment bone reconstruction was associated with significantly higher rates of plate exposure compared to single-segment bone reconstructions (21.2% vs. 6.1%, *p* = 0.041). The institute’s policy was to use hand-bent off-the-shelf reconstruction plates for single-segment reconstructions, while multi-segment reconstructions were plated with PSIs. Significant differences in plate exposure were found between reconstructions with bone using the conventional plate and PSI. (6.5% vs. 25.0%, *p* = 0.028). When comparing plate exposure in reconstructions without bone using the conventional plate and Bookshelf-PSI no significant differences in frequency were reported (30.8% vs. 61.5%, *p* = 0.116).

#### 3.5.3. Plate Fracture

Plate fractures were reported in 12 patients (10.9%). No plate fractures were observed in the PSI or Bookshelf-PSI groups; plate fractures were only observed in the conventional and mini plate groups. Exposed plates did not fracture. Significant difference in plate fracture was found between reconstructions with bone using the conventional plate and PSI. (15.2% vs. 0%, *p* = 0.044). When comparing plate fractures in reconstructions without bone no significant differences in frequency were reported. (23.8% vs. 0%, *p* = 0.066).

#### 3.5.4. Bone Resorption

Bone resorption was reported in 21 patients (19.1%); this resulted in screw loosening in 13 patients (11.5%). Bone resorption was significantly more common in patients without a bone graft (14.6% vs. 32.1%, *p* = 0.012). Screw loosening was significantly more common in patients without a bone graft (7.3% vs. 25.0%, *p* = 0.012). Higher frequencies of bone resorption and screw loosening were observed in reconstructions with bone using PSIs (25.0% and 12.5%) compared to conventional plating (13.0% and 4.3%); this difference was not significant. In reconstructions without bone, the rate of bone resorption and screw loosening was the same.

### 3.6. Plate Removal

Total plate removal was required in 18 patients (16.4%), due to plate exposure, screw loosening, or plate fracture. Plate removal was not registered as a complication, since it was considered a consequence of plate-related complications. It occurred significantly more in patients without a bone graft (*n* = 10/28) compared with patients with a bone graft (*n* = 8/82) (*p* = 0.001). The highest percentage was in reconstructions without bone using hand-bent conventional plating (46.2%). Plate removal occurred after a median time of 375 days (IQR 540), with three outliers of 1435, 1881, and 3452 days. These outliers were likely due to incidental findings after years without a follow-up radiograph.

### 3.7. Guides Not Used

In six patients (5.4%), at least one of the two resection guides was not used either due to mismatch of tumor size at MRI and during surgery (*n* = 5/220) or overestimation (*n* = 1/220) of the tumor on MRI. The performed resection was cut without a guide, and the corresponding fibula was adjusted proportionally to ensure proper fit of the neomandible.

### 3.8. Flap Failure

Flap complications occurred in seven patients (6.4%) and occurred within the first two weeks after surgery. Three fibula-graft, one pectoralis major flap, and one deep circumflex iliac artery flap failed completely (4.5%) and required removal. Two pectoralis major flaps demonstrated partial failure and were debrided.

### 3.9. Local Recurrence

Local recurrence was observed in 21 patients (18.9%). Among the patients with positive bone margins, one patient (1/7) had a local recurrence. Local recurrence rates were higher in cases with closer soft tissue margins: 29% in positive margins (*n* = 9/31), 21.6% in close margins (*n* = 11/52), and 3.7% in clear margins (*n* = 1/28).

### 3.10. Osteotomy Plane Accuracy

The accuracy of the resection planes was evaluated using 155 measurements across five key points per plane, with comparisons made between anterior (n = 78) and posterior (n = 77) planes. Eight resection planes (4.9%), in six patients, were excluded from the analysis, one of two planes in four patients and both planes in two patients. This was because the surgeon chose not to use the cutting guide either due to mismatch of tumor size at MRI and during surgery or overestimation of the tumor on MRI. One case included only a single resection plane, because it involved planned removal of the TMJ. The mean deviation of the central point of gravity was 1.63 mm (SD ± 1.42; range 0.10–5.96 mm, excluding one outlier of 8.96 mm), and was significantly lower in posterior planes (1.32 ± 1.15 mm) than in anterior planes (1.94 ± 1.59 mm; *p* = 0.011). Similar trends were found at the buccal (1.77 mm; range 0.01–9.36 mm, *p* < 0.001), lingual (1.64 mm; range 0.00–7.40 mm, *p* = 0.030), and inferior points (1.98 mm; range 0.07–8.79 mm, *p* = 0.047). No significant difference was observed at the superior point (1.70 mm; range 0.01–6.81 mm, *p* = 0.199). The mean angular deviation was 8.54° (±5.66), with no significant difference between anterior and posterior planes. Resection planes shifted towards the tumor in approximately half of the measurements (47.1–51.6%) (see Table 3).

### 3.11. Mandibular Reconstruction Accuracy

Data from 82 cases were analyzed using linear and angular measurements. The mean absolute difference (MAD) for the intercondylar distance (ICD) was 1.86 mm (SD ± 1.54), for the intergonial distance (IGD) was 2.57 mm (SD ± 1.99), and for the anteroposterior distance (APD) was 2.12 mm (SD ± 1.71). On the operated side, the gonial angle had a MAD of 3.50° (SD ± 2.63), the axial angle was 3.09° (SD ± 3.05), and the coronal angle was 2.83° (SD ± 2.14). On the non-operated side, the gonial angle had a MAD of 2.00° (SD ± 1.61), the axial angle was 2.98° (SD ± 3.12), and the coronal angle was 2.51° (SD ± 2.04). A statistically significant difference was found between the conventional and PSI groups for the IGD (*p* = 0.048), the coronal angle on the operated side (*p* = 0.008), and the gonial angle on the non-operated side (*p* = 0.040) (see Table 4).

### 3.12. Intra-Observer Variation

An intraclass correlation coefficient (ICC) was calculated for the total mandible analysis measurements to assess intra-observer variability. Measurements of 10 patients were repeated after four weeks. All measurements demonstrated excellent reliability.

### 3.13. Inter-Observer Variation

An interclass correlation coefficient (ICC) was also calculated for the total mandible analysis measurements to assess inter-observer variability. Measurements of 10 patients were repeated by a second observer. All measurements demonstrated excellent reliability.

## 4. Discussion

### 4.1. Adherence to Dutch Guidelines

This study assessed adherence to the Dutch national guidelines recommending a care pathway interval (CPI) of a maximum of 30 days and a bone resection margin of at least 5 mm. The median CPI exceeded the recommended threshold, with a median duration of 34.0 days. Furthermore, it was found that 93.7% of bone margins were adequate and soft tissue margin was adequate in 25.2%.

Prolonged CPI has been associated with poorer survival outcomes in head and neck cancer [2,4,5,6]. A recent systematic review reported a strong association between treatment delays and reduced overall survival [2], although some studies found no consistent impact on survival [26,27]. In support of this, Villemure-Poliquin et al. reported that initiating treatment within 30 days improves survival by up to 9% [4]. Additionally, Metzger et al. found a 1.8% increase in mortality risk per day of delay in early-stage disease, an effect not observed in advanced-stage cases [5].

It has been reported that the use of three-dimensional (3D) virtual surgical planning (VSP) may affect CPI. Centers lacking an in-house design 3D-laboratory must rely on external suppliers for design and fabrication, which may take up to three weeks for delivery to the hospital [28]. Although this is not an issue in benign conditions or orthognathic procedures, it can have a substantial effect on the CPI for malignant disease. Knitschke et al. reported an 11-day delay due to imaging, guide fabrication, and coordination with external vendors, though this did not affect resection margins or oncologic outcomes [29]. Similarly, Villarme et al. reported a significant increase in CPI with the use of VSP, with a mean time increase of 13 days, and no effect on oncologic outcome [30]. The current study found that the fabrication lead time of the resection guides and the PSIs was a median of 6.0 days. The UMCG 3D-lab is ISO13485-certified [31] to design plates and guides in-house, while production is performed by commercial suppliers. Digital planning can be completed in one day, while fabrication of the resection guide and PSI performed by external suppliers may need three to five days to produce. The CPI exceeded the national guidelines in several cases, while fabrication times of resection guides and PSIs were short. This suggests that the prolonged timelines do not appear to stem form the 3D-VSP workflow but are influenced by coordination and scheduling delays, supporting its integration without causing significant delays in CPI.

Bone margin status is a key prognostic factor in head and neck cancer, with positive margins reporting a twofold reduction in survival [32]. In a previous study from the same center, no positive bone margins were reported, possibly due to small sample size [1]. In this study, the rate of positive bone margins was notably low (6.3%), suggesting effective surgical planning and execution. Similar results were reported in another 3D-VSP study, with a 4.9% positive margin rate [29], compared to a study that did not use VSP, where positive bone margins are reported at 10.5% [33].

This study found that imaging tended to overestimate medullary bone invasion, which is clinically significant as bone involvement classifies oral squamous cell carcinoma (OSCC) as T-stage IV regardless of tumor size, and is linked to poorer survival [34]. MRI may overestimate tumor size due to chemical shift and inflammation, potentially leading to clinical upstaging and influencing resection planning [35,36]. Noticing these trends might help in cautious reduction in planned bone resection margins resection in certain cases, which could potentially decrease the extent of surgical defects without compromising oncologic safety.

An important finding of this study was that obtaining a clear margin in high volume tumors (>15.0 cc) is challenging. This is clinically relevant as closer margins were associated with a higher recurrence rate in this study. 3D-planning facilitates accurate bony resection, optimization of soft tissue margin clearance remains necessary. Intraoperative techniques like fluorescence imaging and intraoperative PET-CT may assist in improving soft tissue clearance [37,38].

### 4.2. Complication Rate

Another aim of this study was to compare the clinical outcomes of conventional plates and PSI. An overall plate-related complication rate of 39.1% was found, comparable with reported rates by other authors of 25% to 60% [10,11,13,15,17]. The most common complication was plate exposure (21.8%), consistent with other studies which reported ranges of 11% to 30% [10,11,12,13,14,15,16,17] (see Table 5).

With the steadily increasing aging population, an important point to consider is the complication rate of reconstruction without a bone graft. The proportion of patients who are unsuitable for a bone graft is expected to increase, indicating a need for refining reconstructive techniques for this growing cohort. Complications were significantly more frequent in reconstructions without a bone graft, particularly plate exposure. This was also the case for bone resorption, screw loosening, and consequent plate removal.

Larger defects and multi-segment reconstructions showed higher rates of plate-related complications. This is not surprising, as a greater portion of the mandibula must be bridged by multiple segments and a longer osteosynthesis plate, which could experience greater mechanical stress and more muscle insertions removed [39,40].

PSIs were associated with significantly higher rates of plate exposure compared with patients who received conventional plates with bony reconstruction. A similar trend was observed when comparing multi-segment reconstructions to single-segment reconstructions. Comparisons between PSIs and conventional plates should be interpreted with caution since PSIs are typically used in multi-segment reconstruction in the study center, and hand-bent reconstruction plates only in single-segment reconstructions, the observed difference may be due to the complexity and extent of the defect rather than the PSI itself.

Plate exposure was also frequently observed in Bookshelf-PSI reconstructions. A likely explanation of the frequent occurrence of plate exposure when no bone graft is used, is that soft tissues without underlying bone support are more susceptible to mechanical stress and soft tissue contraction. In such cases, the plate may act as a foreign body, triggering a response that leads to plate exposure, even after coverage with soft tissue flaps. Such behavior suggests the need for alternative plate designs or improved plate surface characteristics, particularly in reconstructions without bony support.

PSIs eliminate the need for intraoperative manual bending, which results in residual mechanical stress and weak points in the plate material, as they are fabricated to fit the patient’s anatomy [8]. This improves fit, bone-to-plate contact, and biomechanical stability, reducing the risk of deformation or failure under load [39,40]. No plate fractures occurred in the PSI and the Bookshelf-PSI group, consistent with other studies [10,39]. Plate fractures did occur in cases when conventional plating without a bone graft was used and often lead to plate removal. Plate fractures in the conventional group did not lead to removal, as the bone graft provided enough structural support once osseointegrated. Removal was often required due to screw loosening caused by stress shielding-induced bone resorption or plate exposure [18].

### 4.3. Accuracy of Resection Plane and Mandibular Reconstruction

The final aim of this study was to evaluate the accuracy of both the resection plane and the total mandible reconstruction. A mean absolute difference (MAD) of 1.63 mm (±1.42) was observed for the central point of gravity of resection planes. The MAD was 1.86 mm (±1.54) for intercondylar distance, 2.57 mm (±1.99) for intergonial distance, and 2.45 (±2.35) for anteroposterior distance. Deviations from the virtual plan are common and can arise at multiple stages of execution [41]. The observed deviations from the surgical plan were minimal and fall within the range commonly reported in mandibular resection and reconstruction, suggesting limited clinical relevance [40,41,42,43,44,45,46].

In this study, posterior resections showed significantly lower deviation, in contrast to reports of higher accuracy in anterior resections [45,46]. This may be due to clearer posterior anatomical landmarks, such as the mandibular angle or coronoid notch, which aid guide positioning [41,45]. Limited access to the proximal segment may restrict freedom of movement, resulting in less deviation from the plan. In contrast, with anterior resections, particularly in edentulous patients, it can be difficult to precisely position the cutting guide due to the conical shape of the mandible, narrow width, and lack of clear landmarks [41].

Larger defects are linked to greater deviations [44], as each additional segment and osteotomy increases cumulative error. This study demonstrated significant differences in intergonial distance and the operated coronal angle between the conventional plates and PSI, with PSIs showing less deviation. This may be explained by their anatomical fit, which reduces flaring of posterior segments and limits intraoperative movement during fixation, leading to less deviation. These findings support the use of PSIs in multiple segment reconstructions, as implemented in the clinical workflow of the study center.

In six cases in this study, the resection guides were not used due to discrepancy between expected tumor size and the actual tumor size which made pre-planned margins inadequate. Current resection guides cannot accommodate significant deviations from the surgical plan. This can be considered a potential limitation of 3D-VSP, and surgeons should still be prepared to adapt the surgical plan in those situations. Although incorporating a backup plan into the cutting guide has been proposed [41,47], routine implementation may not be a cost-effective strategy. Adhering to treatment timelines remains essential to minimize potential tumor progression, though the rate of tumor growth is unpredictable and cannot be foreseen in advance.

### 4.4. Limitations

The first and foremost limitation of this study is its retrospective design and lack of randomization limits control over variables and standardization across cases. Secondly, certain cases were excluded in the accuracy analysis due to unavailable data, which may introduce selection bias. As the missing data were unrelated to case complexity, we believe the impact of this limitation on our findings is unlikely to be significant. Additionally, there was heterogeneity in postoperative imaging. About one-quarter of postoperative scans were lower resolution CT, potentially reducing segmentation accuracy and affecting resection plane assessment. Finally, manual measurements were used, although efforts were made to ensure objectivity and reproducibility, the process is time-consuming and may introduce variability.

Future studies should study automated segmentation with artificial intelligence may simplify workflows and ensure consistency. Strategies to minimize soft tissue complications are needed, as these frequently lead to re-intervention. Additionally, new implants materials that reduce stress shielding and plate exposure should be investigated [18,19].

## 5. Conclusions

The care pathway interval (CPI) was frequently exceeded and associated with coordination and scheduling delays. Fabrication times for resection guides and PSIs were short, suggesting that extended CPI are associated with operational factors rather than the 3D virtual surgical planning (VSP) workflow. The current clinical workflow using 3D-VSP with multi-modality imaging has proven successful in achieving adequate bone margins. Soft tissue margin clearance remains a challenge but is typically not a part of 3D planning. Shortening the CPI and intraoperative tumor visualization are key steps toward improving overall oncologic outcomes.

The absence of a bone graft is particularly associated with plate-related complications. Plate exposure was the most frequent complication and is a problem in both bony reconstructions and non-bony reconstructions. PSIs were associated with more plate exposure than conventional plating but showed mechanical superiority, as no plate fractures occurred. The key to long-term success depends on soft tissue preservation, material surface optimalisation, and maintaining bone stability around the osteosyntheses plates.

This study highlights that three-dimensional virtual surgical planning achieves high surgical accuracy. Clinically relevant deviations can still occur but are low in numbers. Continued refinement of both preoperative planning and intraoperative execution is essential to further optimize resection and reconstruction.

## Figures and Tables

**Table 1:** Patient demographics, tumor staging, and surgical details.

		All (111)				
Gender (%)	Male	51 (45.9%)				
	Female	60 (54.1%)				
Age (years)	Mean (SD)	66.7 (±13.9)				
Risk Factors	Smoking	69 (62.2%)				
	Alcohol	55 (49.5%)				
Status (%)	Alive	54 (48.6%)				
	Dead	57 (51.4%)				
Follow-up (months)	Mean (SD)	59.8 (±144.8)				
T (%)		Clinical	Pathological	Cohen’s K 0.272	Weighted-K 0.367	*p*-value * 0.338
	T1	8 (7.2%)	8 (7.2%)			
	T2	19 (17.2%)	25 (22.5%)			
	T3	5 (4.5%)	2 (2.4%)			
	T4	79 (71.2%)	76 (68.5%)			
N (%)	N0	55 (49.5%)	78 (70.3%)	0.217	0.281	<0.001
	N1	18 (16.2%)	12 (10.8%)			
	N2	36 (32.4%)	16 (14.4%)			
	N3	2 (1.8%)	5 (4.5%)			
M (%)	M0	111 (100%)	111 (100%)			
	M1	0	0			
Location	Left	41 (36.9%)				
	Right	46 (41.4%)				
	Front	24 (21.7%)				
Brown Classification	I	38 (34.2%)				
	II	49 (44.1%)				
	III	23 (20.7%)				
	IV	1 (0.9%)				
Used flap	Fibula	81 (73.0%)				
	Pectoralis	25 (22.5%)				
	major					
	Other	5 (4.5%)				
Number of segments	0	29 (26.1%)				
	1	49 (44.1%)				
	2	26 (23.4%)				
	3	7 (6.3%)				
Used plate	Conventional	59 (53.2%)				
	Miniplate	12 (10.8%)				
	PSI	26 (23.4%)				
	Bookshelf-PSI	13 (11.7%)				
Adjuvant therapy	Radiotherapy	71 (64.0%)				
	Chemotherapy	19 (17.1%)				

Wilcoxon signed rank test between clinical and pathological *.

**Table 2:** Histopathological features.

	All (111)				
Bone margin					
Yes	104 (93.7%)				
No	7 (6.3%)				
Soft tissue margin					
Positive	31 (27.9%)				
Close	52 (46.8%)				
Clear	28 (25.2%)				
Tumor volume in cc (mean (SD))	11.2 (±12.0)				
Tumor diameter in mm (mean (SD))	28.6 (±14.6)				
Tumor depth of invasion (mean (SD))	13.0 (±9.9)				
	Pathological	Radiological	Cohen’s K	Weighted-K	*p*-value *
Bone invasion Cortex Medulla Perineural invasion Lymphovascular invastion	75 (67.6%) 41 (51.9%) 34 (48.1%) 39 (35.1%) 18 (16.2%)	77 (69.4%) 23 (27.0%) 54 (73.0%)	0.267	0.338	0.013

Wilcoxon signed rank test between pathological and radiological *.

**Table 3:** Accuracy of the resection plane.

Measurements	All Planes (155)	Posterior Plane (78)	Anterior Plane (77)	Plane Shift (155) *	*p*-Value **
Center of gravity (mm)	1.63 (±1.42)	1.32 (±1.15)	1.94 (±1.59)	73 (47.1%)	0.011
Buccal (mm)	1.77 (±1.77)	1.23 (±1.32)	2.32 (±2.00)	73 (47.1%)	<0.001
Lingual (mm)	1.64 (±1.47)	1.42 (±1.29)	1.86 (±1.61)	79 (51.0%)	0.030
Superior (mm)	1.70 (±1.39)	1.54 (±1.25)	1.87 (±1.51)	80 (51.6%)	0.199
Inferior (mm)	1.98 (±1.90)	1.71 (±1.77)	2.26 (±2.00)	74 (47.7%)	0.047
Angle (°)	8.54 (±5.66)	8.80 (±5.62)	8.27 (±5.72)		0.533

Measurements are noted in absolute mean (SD) difference in mm. Towards tumor * Wilcoxon signed rank test comparing resection planes **.

**Table 4:** Accuracy of mandibular reconstruction.

Measurements	All Patients (82)	Conventional (41)	PSI (22)	*p*-Value *
Intercondylar distance (mm)	1.86 (±1.54)	1.97 (±1.83)	1.61 (±1.17)	0.757
Intergonial distance (mm)	2.57 (±1.99)	2.78 (±2.07)	1.65 (±1.07)	0.048
Anterio-posterior distance (mm)	2.21 (±1.71)	1.98 (±1.29)	2.44 (±2.46)	0.914
Operated side				
Gonial Angle (°)	3.50 (±2.63)	3.00 (±1.54)	3.51 (±2.76)	0.419
Axial Angle (°)	3.09 (±3.05)	2.75 (±2.11)	3.96 (±3.27)	0.231
Coronal Angle (°)	2.83 (±2.14)	3.35 (±2.25)	1.89 (±1.41)	0.008
Non-Operated side				
Gonial Angle (°)	2.00 (±1.61)	1.57 (±1.43)	2.51 (±1.89)	0.040
Axial Angle (°)	2.98 (±3.12)	3.28 (±3.21)	3.39 (±3.27)	0.719
Coronal Angle (°)	2.51 (±2.04)	2.68 (±2.12)	2.20 (±1.72)	0.419

Measurements are noted in absolute mean (SD) difference. Mann–Whitney U test between conventional and PSI *.

**Table 5:** Plate-related complications—overview other authors.

	N	Plate-Related	Plate Exposure	Plate Fracture	Screw Loosening	Bone Resorption	Plate Removal
Present study	110	39.1%	21.8%	10.9%	11.8%	19.1%	16.4%
Kreutzer [10]	83	47.0%	20.5%	0.0%	2.4%	12.5%	32.5%
Dean [11]	111	26.1%	14.4%	2.7%	2.7%	2.7%	18.9%
Eskander [12]	515	-	15.0%	-	-	-	-
Rendenbach [13]	128	60.2%	21.9%	-	7.0%	-	31.3%
Van Gemert [14]	79	-	11.3%	5.1%	-	5.1%	18.9%
Chang [15]	219	25.4%	15.6%	13.7%	-	-	-
Davies [16]	94	-	30.0%	-	12.8%	-	-
Walia [17]	266	30.0%	26.7%	2.6%	-	9.0%	18.0%

## Data Availability

The original contributions presented in the study are included in the article, further inquiries can be directed to the corresponding author.

## Abbreviations

The following abbreviations are used in this manuscript:

OSCC	Oral squamous cell carcinoma
CPI	Care pathway interval
3D-VSP	Three-dimensional virtual surgical planning
UMCG	University Medical Center Groningen
PSI	Patient-specific implant
DICOM	Digital Imaging and Communications in Medicine
ICD	Intercondylar distance
IGD	Intergonial distance
APD	Anteroposterior distance
SD	Standard deviation
IQR	Interquartile range
ICC	Intraclass correlation coefficient
MAD	Mean absolute difference
3D	Three-dimensional
